# An assessment of the impact of climate adaptation measures to reduce flood risk on ecosystem services

**DOI:** 10.1007/s10980-012-9715-6

**Published:** 2012-02-11

**Authors:** Peter H. Verburg, Eric Koomen, Maarten Hilferink, Marta Pérez-Soba, Jan Peter Lesschen

**Affiliations:** 1 Institute for Environmental Studies, VU University Amsterdam, de Boelelaan 1087, 1081 HV Amsterdam, The Netherlands; 2 Faculty of Economics and Business Administration, VU University Amsterdam, de Boelelaan 1105, 1081 HV Amsterdam, The Netherlands; 3 ObjectVision B.V., de Boelelaan 1087, 1081 HV Amsterdam, The Netherlands; 4 Alterra, PO Box 47, 6700 AA Wageningen, The Netherlands

**Keywords:** Climate change adaptation, Integrated spatial modelling, Land use, Ecosystem services, Flood risk, Soil protection

## Abstract

Measures of climate change adaptation often involve modification of land use and land use planning practices. Such changes in land use affect the provision of various ecosystem goods and services. Therefore, it is likely that adaptation measures may result in synergies and trade-offs between a range of ecosystems goods and services. An integrative land use modelling approach is presented to assess such impacts for the European Union. A reference scenario accounts for current trends in global drivers and includes a number of important policy developments that correspond to on-going changes in European policies. The reference scenario is compared to a policy scenario in which a range of measures is implemented to regulate flood risk and protect soils under conditions of climate change. The impacts of the simulated land use dynamics are assessed for four key indicators of ecosystem service provision: flood risk, carbon sequestration, habitat connectivity and biodiversity. The results indicate a large spatial variation in the consequences of the adaptation measures on the provisioning of ecosystem services. Synergies are frequently observed at the location of the measures itself, whereas trade-offs are found at other locations. Reducing land use intensity in specific parts of the catchment may lead to increased pressure in other regions, resulting in trade-offs. Consequently, when aggregating the results to larger spatial scales the positive and negative impacts may be off-set, indicating the need for detailed spatial assessments. The modelled results indicate that for a careful planning and evaluation of adaptation measures it is needed to consider the trade-offs accounting for the negative effects of a measure at locations distant from the actual measure. Integrated land use modelling can help land use planning in such complex trade-off evaluation by providing evidence on synergies and trade-offs between ecosystem services, different policy fields and societal demands.

## Introduction

Evidence and awareness of climate change has led to an increasing need to adapt our use of land and other resources to limit risks and vulnerabilities that originate from global change (Adger et al*.* 2005; Foley et al*.* 2005). Changes in precipitation and temperature give rise to changes in the hydrology of river systems. At the same time human-induced land use changes, e.g. deforestation of upstream catchments, lead to changes in run-off conditions. The combined effects of land use change and climate change may lead to increased flood risk and changes in ecosystem service delivery (Bouwer et al*.* 2010; Hurkmans et al*.* 2009; Metzger et al*.* 2008). Flooding of rivers upon peak discharge is a natural process. However, the increasing population densities in floodplain areas together with increased assets located in flood-prone regions leads to an ever-increasing vulnerability of people and financial damage upon flooding (Barredo 2009; de Moel et al*.* 2011). Given these conditions, adaptation and mitigation strategies to reduce flood risk and exposure to flooding are developed (Biesbroek et al*.* 2010). Many measures for adaptation to climate change are related to changes in planning and management of land use (Dawson et al*.* 2011). Measures can include restrictions on residential and commercial functions in areas sensitive to flooding, reforestation of sloping land in the upper part of catchments and the allocation of retention areas. The claims made on land resources for such measures may, especially in densely populated delta regions, conflict with other claims for land, e.g. those for food and energy production, for urban development or for biodiversity conservation. Planning of adaptation policies, therefore, requires a careful analysis of possible tradeoffs of such measures in other domains. At the same time it is expected that adaptation measures not only contribute to climate and water regulation, but have synergistic effects on other ecosystem services. Conservation and restoration of riverine wetlands does not only benefit flood regulation but also provides carbon sequestration and habitat functions (Vos et al*.* 2010). A careful choice of the adaptation measures fitted to the context of a specific region will benefit other ecosystem services while avoiding unintended tradeoffs. Insight into the possible synergies and tradeoffs may help the design of more integrated policy packages that can be implemented at the appropriate institutional levels (Helbron et al*.* 2011).

**Fig. 1:**
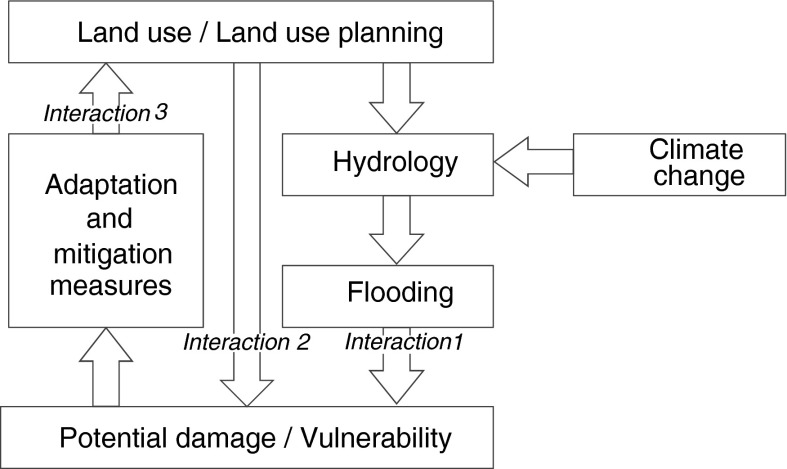
Interactions between land use and the vulnerability/damage as result of changes in flood occurrence

Land use and land use planning play a critical role in the evaluation of possible strategies to adapt to the consequences of climate change and increased flooding in particular (Fig. 1). Land use change is a driver of changes in the hydrological system (interaction 1 in Fig. 1) and it influences the potential damage and vulnerability of people and assets (interaction 2). However, at the same time, land use and land use planning are a means of adaptation (interaction 3). Assessments of land use change scenarios have provided insight in the evolution of future land use and related impacts on ecosystem services through the simulation and analysis of exploratory scenarios (Kienast et al*.* 2009; Rounsevell et al*.* 2006; Sohl et al*.* 2007; Verburg et al*.* 2010). However, to further assist planning and implementation of adaptation policies a more targeted scientific approach is needed. Perrings et al. (2010) argues that scientific assessments in the field of ecosystem services and biodiversity should aim at evaluating the impacts of specific (combinations of) measures rather than focus on broad overarching scenarios. The authors argue that explicit attention should be given to the identification of potential synergies and tradeoffs of such measures on ecosystem services and biodiversity.

This paper intends to take such an approach by analyzing the land use consequences of a policy package of adaptation measures for the territory of the European Union. The results are used to analyze to which extent these measures have synergetic effects on biodiversity and ecosystem conservation.

## Methodology

### Overall approach

**Fig. 2:**
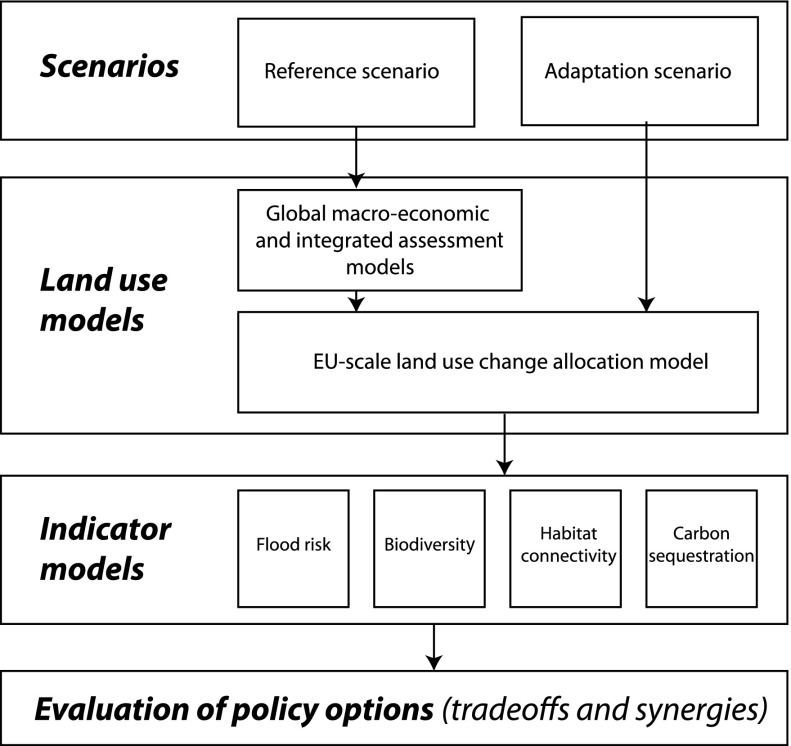
Overview of the methodology

To analyze the land use consequences of adaptation measures two scenarios are analyzed for a 30 year period (2000–2030). The first scenario is a reference scenario that represents a continuation of ongoing economic and demographic trends and includes a number of important ongoing policy developments affecting land use. The second scenario is based on the same macro-level assumptions but includes a package of spatial policies that are related to adaptation measures. Both scenarios were evaluated with a series of models that translate scenarios of macro-economic change to spatial patterns of land use change. Finally four indicators of impacts on ecosystem services were calculated: flood risk, carbon sequestration, biodiversity and habitat connectivity. Based on these indicators the tradeoffs and synergies of the adaptation measures are evaluated. Figure 2 provides an overview of the methodology.

### Scenarios

For the development of the reference scenario use is made of the well-known B1 scenario of IPCC-SRES (IPCC 2000) and elaborated for the European conditions by Westhoek et al. (2006). The scenario accounts for global scale drivers influencing European land use like:

- increasing food and feed demand in emerging countries, i.e. the BRIC countries (Brazil, Russia, India and China);
- changing trade regimes because of increasing competitiveness of Asian and Latin-American regions;
- changing environmental constraints because of resource scarcity and climate change (following climate change calculations by the IMAGE model (Bouwman et al*.* 2006);
- demographic changes.

The B1 scenario as specified by Westhoek et al. (2006) includes a number of important policy developments that correspond to ongoing changes in policies, such as the reform of the European Union Common agricultural policy. As compared to the assumptions of the other scenario storylines prepared before the financial crisis, it includes for Europe a modest economic growth which is realistic for the economic conditions after the economic crisis of 2007–2008. Some of the specific European environmental policies in this scenario were modified from the original description by Westhoek et al. (2006) to better match the current policy context. As such, it may be interpreted as a business-as-usual type of scenario. An overview of the most important socio-economic assumptions and key characteristics for the EU is provided in Table 1.

**Table 1:** Reference scenario socio-economic assumptions and key characteristics for the EU

Aspect	Scenario assumptions
Population EU-27 in 2030	500 million
Population change since 2000	4%
EU-15 GDP yearly growth	1.3%
EU-12 GDP yearly growth	3.4%
Trade of agricultural products	Export subsidies and import tariffs phased out. Slight increase in non-tariff barriers
Product quota	Phased out; abolished by 2020
Farm payments	Fully decoupled and gradually reduced (by 50% in 2030)
Intervention prices	Phased out; abolished by 2030

In addition to these macro-level conditions in terms of economic change, trade agreements, the common agricultural policy and demography, also policies that directly affect the spatial patterns of land use are included in the scenario specification. The reference scenario contains a number of current spatial EU policies. Important examples are the Less Favoured Areas (LFA) support (compensation to farmers in regions with constraints for agricultural use), and current protected nature areas (including the EU defined Natura 2000 areas, forests and other natural areas). In this way the reference scenario offers business-as-usual baseline conditions that allow a proper assessment of the impacts of policy alternatives.

An alternative policy scenario was developed to evaluate the spatial planning of land use for the conservation of soil and regulation of water in connection to climate change. The macro-level socio-economic developments (Table 1) and climate change assumed were identical to the reference scenario. This scenario is based on policy themes that are currently being discussed within the European Union (Table 2). The specification of the scenario was achieved as a joint process between modellers and policy makers at the European Commission in Brussels. The scenario options were elaborated in three interactive steps. This process ensured a good correspondence between the scenario assumptions and the ongoing policy discussion. In the first step a number of broad issues and policy themes were identified that should be addressed in the scenarios. This list was elaborated with qualitative descriptions of the policy ambitions and actions possible within this theme based on policy documents and public discussion. The final step accounted for the translation of these qualitative descriptions into settings of the model. The modellers made a first proposal which was explained to the policy makers. A lack of clarity in the specification of the measures was revealed and in a number of cases the policy makers were requested to specify more clearly the actual functioning of the proposed policy mechanisms. This resulted in a jointly agreed set of scenario conditions that could easily be implemented in the model framework. For all identified policy themes both the reference scenario and the policy alternative were specified. A selection of the most important qualitative descriptions related to climate adaptation is provided in Table 2. A full overview of all scenario specifications and model settings is provided by Pérez-Soba et al. (2010).

**Table 2:** Overview of the current spatial policy ambition level incorporated in the reference scenario and the more ambitious policies in the policy alternative

Policy theme	Current ambition level	Policy alternative
Flood damage reduction	Current national and EC (Flood directive) policies based on current flooding statistics	Discouraging urbanisation in areas that are likely to become more flood prone due to climate change. Promotion of extensive agriculture and nature in these areas
Restore water balance (limits probability on floods and droughts)	Water framework directive	Discourage urbanisation and promote forest, nature and extensive forms of agriculture (grassland) in upstream parts of catchment areas
Protection permanent pasture	Some incentives to avoid conversion of permanent pasture; maximum decrease in total permanent pasture area	Strict protection of permanent pasture areas.
Protection peatland	No policies	Land conversions in peaty areas are not allowed
Soil protection	Thematic strategy for soil protection communication	Spatial planning to promote more compact forms of urbanisation to reduce soil sealing
Erosion prevention	Limited incentive to convert arable land on erosion sensitive places to grassland and forestry (current Common agricultural policy measure)	Strong incentive to convert arable land on erosion sensitive places to grassland and forestry

The adaptation measures accounted for different aspects of the relation between land use and vulnerability to flooding. The Directive 2007/60/EC on the assessment and management of flood risks (EC 2007) requires member states to assess if water courses and coast lines are at risk from flooding, to map the flood extent, assets and humans at risk in these areas, and to take adequate and coordinated measures to reduce this flood risk. It also requires member states to take into consideration long-term developments, including climate change, as well as sustainable land-use practices in the flood risk management cycle addressed in this Directive. One of the measures we have accounted for in this scenario is more regulation of land use planning in flood prone areas. Flood prone areas are defined by those areas in which a minimum of 25% of the 1 km^2^ pixel is designated as experiencing an inundation of 50 cm or more in a 100 year flood event according to a map prepared by the EC Joint Research Center (Barredo et al*.* 2007). In those areas no new urban land use is allowed while extensive agriculture (grassland) and nature are favoured above intensive agriculture. Such measures are especially aimed at reducing the potential damage of flood events rather than reducing the flood risk itself. Increased variability in precipitation and higher summer temperatures will, most likely, also lead to more pronounced water shortages in summer time. This is likely to impact, for example, agricultural practices and shipping on the major rivers. In recognition of the acuteness of the water scarcity and drought challenges in Europe, the European Commission adopted a Communication addressing the challenge of water scarcity and droughts in the European Union (COM/2007/414). The Communication provides a fundamental and well-developed first set of policy options for future action, within the framework of EU water management principles, policies, and objectives. To implement such notions it is suggested to promote the storage of rainwater in the hydrological system (surface and groundwater) in upstream areas to secure a more constant delivery of water to river systems. This policy has the potential of reducing the peeks in river discharge and thus limits the chance of flooding. As such it increases the ecosystem service of water supply and regulation. The policy objective of increasing the amount of rainwater retention and infiltration can be implemented in the model through the promotion of nature, forest and extensive forms of agriculture in upstream areas. Upstream areas are, arbitrarily, delineated by the upper 10% of the height range in each catchment area.

Also synergies between climate change adaptation and other policies are considered. Policies to avoid the conversion of permanent pasture not only favour carbon sequestration (Schulp et al*.* 2008) but also lead to lower peak flows due to reduced run-off in sloping areas. Similarly it is assumed that in the policy alternative land conversions on peat soils are not allowed given the role of these soils in regulating water quantity.

In line with the Thematic strategy for soil protection of the European Commission, soil sealing, leading to fast run-off after precipitation, is prevented by promoting compact urbanization in land use planning. In mountainous areas incentives of the Common agricultural policy to convert arable land on erosion sensitive slopes to forest or grassland are assumed to be reinforced. The spatial representation of erosion sensitive locations is based on a calculation of current erosion risk given slope, climate and soil conditions following the Universal Soil Loss Equation (Wischmeier 1976).

### Land use modelling

The methodology for assessing land use changes is based on a multi-scale, multi-model approach that integrates the economic, demographic and environmental drivers of land change in a consistent modelling framework described by Verburg et al*.* (2008). Global scale drivers of land use change originating from changes in demography, consumption patterns, economic development, trade and climate change are analyzed with the combined application of the global economy model LEITAP and the global integrated assessment model IMAGE. A detailed description of the interaction between these two models is provided by van Meijl et al*.* (2006) and Eickhout et al*.* (2007). These global scale models provide output in terms of changes in agricultural area (distinguishing arable land and grassland) at the level of individual countries within the European Union. These changes in agricultural area are integrated with claims from the urban/industry sectors which are based on simple analysis of overall relations between urban area, population and GDP using the scenario specific demographic and economic projections. Land cover areas at a national scale are input to the land allocation model. The land allocation model translates the national scale land areas to a 1 km^2^ grid. The model distinguishes arable land, irrigated arable land, permanent crops, grassland, recently abandoned agricultural land, scrubland, forest, build-up land, and a number of smaller classes that are assumed to be more or less static in time. Based on the thus derived land cover maps a number of indicators for the impacts of land use changes can be calculated. The core of the modelling framework including the land allocation model and the indicator models are integrated into a consistent modelling interface called the CLUE-Scanner. The land allocation model is the Dyna-CLUE model (Verburg and Overmars 2009) using the numerical algorithms of the Land Use Scanner model (Koomen et al*.* 2008).

The translation of the national level changes in agricultural area from the LEITAP model to input of the Dyna-CLUE model requires a number of corrections to ensure consistency between the models. While LEITAP is based on agricultural statistics the Dyna-CLUE simulations are based on land cover data derived from CLC2000. Large differences in agricultural areas between the two data sources are the result of differences in definition, observation technique, data inventory bias etc. (Verburg et al*.* 2009b; Verburg et al*.* 2011). To some extent these difference can be corrected as they relate to differences in definition of land cover classes. Absolute changes in agricultural area in LEITAP are corrected for known, structural, differences in representation and then serve as input to the Dyna-CLUE model. The net change in agricultural and urban area will determine the overall area left for semi-natural land use types and forestry. From the IMAGE model climate change data are used as one of the location factors considered in the Dyna-CLUE model. Changes in climate, resulting from the IMAGE model calculations, are at a coarse spatial resolution (50 × 50 km) and are downscaled to 1 × 1 km and superimposed on the more detailed Worldclim data (Hijmans et al*.* 2005) for use in the simulations.

The Dyna-CLUE model is a recent version of the CLUE model (Verburg et al*.* 1999; Verburg et al*.* 2002; Verburg and Overmars 2009). CLUE is one of the most used land allocation models globally and is highly applicable for scenario analysis (Pontius et al*.* 2008). The use of the model in many case studies at local and continental scale by different institutions worldwide (e.g. (Castella et al*.* 2007; Wassenaar et al*.* 2007) has proven its capacity to simulate a wide range of scenarios and provide information for indicator models. The land allocation procedure allocates for each pixel the land cover type with the highest local suitability at that location, constrained by the macro-level demand for the land cover types, the land use history and a set of rules that represent spatial restrictions (e.g. nature reserves). At the same time autonomous changes in land cover can occur through re-growth of natural vegetation given the location specific vegetation growth rates (Verburg and Overmars 2009). Suitability maps that define the specific suitability for each land use type are based on empirical analysis of relations between location of land use and a set of socio-economic and physical properties. For example, the European soil map is translated into functional properties such as soil fertility and water retention capacity. In addition to the soil map a set of approx. 100 factors that range from accessibility to bio-physical properties is considered as potential location factors. A full list of factors considered can be found in Verburg et al. (2006). The suitability at specified locations can be modified as result of assumed policy incentives. Subsidies offered to farmers that compensate for less favourable conditions in marginal areas can raise the suitability for agricultural use at these locations. Taxes on specific activities reduce the suitability. Other scenario conditions are implemented through rules or restrictions on specific land cover conversions in delineated areas.

### Indicator models

Four indicator models were selected in this paper to evaluate the effects of land use changes on indicators connected to the provision of a number of ecosystem services. These indicators only represent a small fraction of the full range of ecosystem services provided in the region. The selection represents services closely connected to other strategies for adaptation and mitigation of climate change: carbon sequestration and biodiversity.

The flood risk indicator highlights the urban areas within the potential flooding zone that are newly developed since 2000. New urban land cover identified by the land allocation model is overlaid with a map of future flood-prone areas (100 year return period) under conditions of climate change. This assessment of potential river-flood risk does not incorporate the conditions of flood defence systems and the effects of upstream land use change on flood occurrence. Therefore, the indicator is especially meant to highlight those areas where new assets become exposed to flood risk. Flood risk from the sea is not included in the analysis.

The second indicator used in this paper is an indicator of carbon sequestration. This indicator is based on a carbon bookkeeping approach that takes into account effects of soil and forest age on carbon stock changes. Emission factors are specified by individual countries and land cover types to account for differences in farming practice and ecosystem function across Europe. Details of the indicator are described by Schulp et al. (2008).

Two indicators are designed to capture the impacts of land use change on biodiversity at the spatial and thematic resolution of the land use modelling results. The first indicator is a measure of the suitability of the habitat for maintaining biodiversity while the second indicator aims to provide a measure of the connectivity of the habitats. Both indicators represent different aspects of habitat quality.

The biodiversity indicator is a Mean Species Abundance (MSA) index which is derived from land use, land use intensity (agriculture and forestry), nitrogen deposition, spatial fragmentation, infrastructure developments and policy assumptions on high nature value (HNV) farmland protection and organic agriculture. The methodology used is based on the GLOBIO3 approach initially developed for biodiversity assessments at a global scale (Alkemade et al*.* 2009), but refined for application at the level of Europe (Verboom et al*.* 2007). The indicator provides an approximation of the land use related changes on biodiversity. The spatial and thematic resolution is not sufficient to discern actual habitats and capture detailed ecological processes. Instead, the above mentioned factors are used to indicate the pressures that species abundance is facing as result of the human impacts on the natural system. The index ranges from 0 to 100, and represents the species abundance compared to species abundance in the natural system without human disturbances. This index of biodiversity has clear limitations and the results do not provide a precise, local account of biodiversity (Trisurat et al*.* 2010). It does, however, provide a broad overview of the impacts of land change on biodiversity and allows for the comparison between the current and different future situations.

The last indicator measures the connectivity of individual patches of natural area. This indicator assesses the difficulty to reach the nearest larger sized habitat from smaller habitats based on the land use allocation results. It offers an approximation of the connectivity of the landscape for species and the viability of smaller habitats within the landscape matrix. The difficulty to reach other habitats is differentiated between land use types, assuming a high resistance of urban and arable areas for the migration of species, a medium to low resistance of permanent grassland areas and a low resistance of small patches of (semi-) natural area. The overall connectivity of an area is assessed by calculating the average resistance (or travel time) to reach the larger patches of natural vegetation from the smaller patches within a neighbourhood or administrative region. As the indicator is not including information on the quality of different land use types, it only offers an indication of the potential coherence of possibly valuable natural areas. The indicator has been defined in such a way to be as much as possible independent of the area of natural land use types in the region and solely capture the spatial arrangement. Therefore, also areas with a relatively small area of nature may still have a good connectivity if the green infrastructure is well-developed. Alternative indicators for landscape connectivity, such as the frequently used proximity indicator (Gustafson and Parker 1994), are not sufficiently sensitive to the data used at the spatial and thematic resolution of this analysis.

## Results

**Fig. 3:**
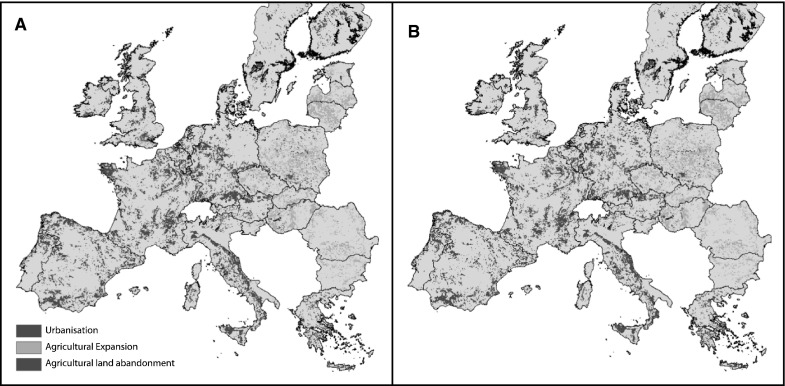
Main land use change processes over the period 2000–2030 for the reference scenario **A**, and the policy alternative focussed on adaptation measures **B**

Figure 3 shows a generalized overview of the results of the land use simulations for the reference scenario and the policy alternative. The original land use modelling results for the period 2000 to 2030 have been summarized by the dominant conversion processes. The overall distribution of changes across the European countries is a direct result of the macro-economic global scale models whereas the spatial patterns within the countries are a result of the spatial allocation procedure. It is obvious that the overall pattern of change is similar for both simulations: the land use areas for individual countries were kept similar in both simulations; the measures were assumed to only affect the spatial patterns. The macro-economic models describe an overall trend of continued abandonment of marginal agricultural lands in Western Europe and some expansion of agricultural land in Eastern Europe and some localized areas in e.g. Spain. To some extent this expansion of agricultural land in Eastern Europe takes place at the cost of fallow land and small patches of (semi-)natural land within the main agricultural areas. However, in some cases the model also predicts expansion of agriculture at the cost of forest areas, especially in the Baltic countries and Poland. The results for deforestation are questionable for some countries, e.g. in Poland more than 80% of the forest is state-owned (Bartczak et al*.* 2008). Deforestation may be a more important issue for countries that have experienced expropriation in the 1950s and where the forest has been transferred back to the initial owners in the 1990s, leading to very small, fragmented ownership structures which favour deforestation. Both the macro-economic models and land allocation model do not include information on the tenure status of land resources and may therefore overestimate the potential for land conversions in eastern Europe. The areas identified as likely locations of agricultural abandonment correspond with areas that have frequently been mentioned as areas at risk of marginalization and in which land abandonment processes have sometimes been ongoing for the last 50 years (MacDonald et al. 2000; Falcucci et al. 2007).

The maps indicate that the adaptation measures are not likely to influence the overall patterns of major land change processes in Europe in the coming decades. Land abandonment will still be concentrated in the most marginal areas and urbanization will take place in the already heavily urbanized regions. However, the adaptation measures will influence land change processes at selected locations and alter regional patterns of conversions. When analyzing the results in more detail the implications of the adaptation measures on land change patterns become apparent.

**Fig. 4:**
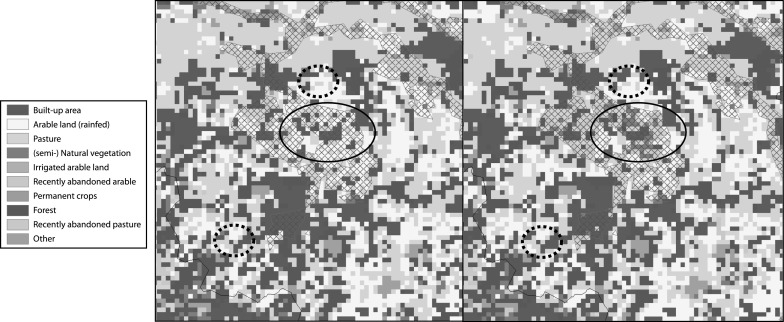
Simulated land use maps for 2030 for an area in The Netherlands (surrounding Eindhoven) for the reference scenario (*left*) and the policy alternative (*right*); areas marked in blue indicate flood prone areas. The ellipse indicates an area where less urbanization occurs in the flood prone area in the policy alternative while the dashed circle indicates more urbanization outside the flood prone area

An example of such regional differences in land use configuration resulting from the measures in the policy alternative is provided in Fig. 4. As a result of new urbanization, the urban area located on flood prone land has increased in the reference scenario while new urbanization has taken place outside the flood prone area in the policy alternative. This example directly indicates that the differences between the scenarios are not restricted to the areas where the policies are actually aimed at. The demand for urban area will be fulfilled elsewhere leading to spatial tradeoffs. In Fig. 4 it can also be seen that, consistent with the specification of the policy alternative, intensive agricultural use in flood prone areas has been abandoned and replaced with semi-natural vegetation.

**Fig. 5:**
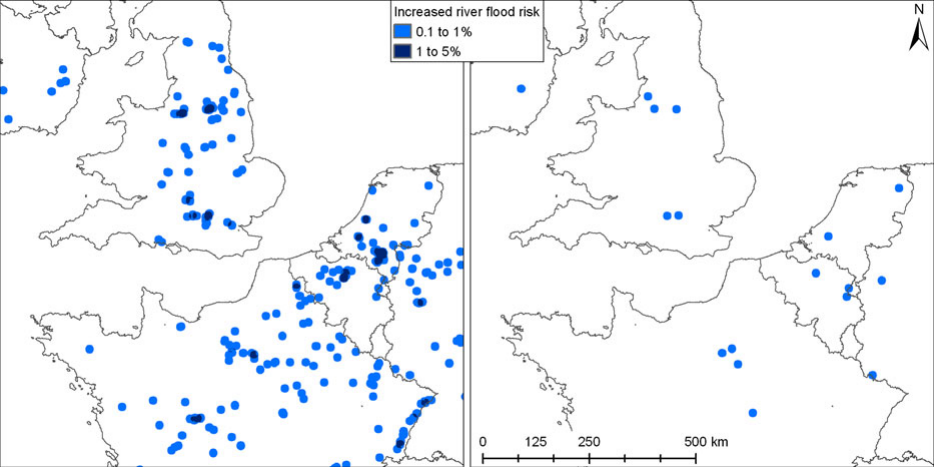
Increased river flood risk over the 2000–2030 period in reference scenario (*left*) and policy alternative (*right*). Risk is expressed here as percentage new urban area of the total land area within a 10 km circular neighborhood prone to river floods that have a statistical return period of occurring once every 100 years under future climate conditions

The flood risk indicator shows the success of the spatial policies in reducing the exposure to potential flooding (Fig. 5). Without these spatial policies it is likely that new urban areas will appear in flood prone areas in all major delta regions of Europe. In total 599 km^2^ of new urban area is located in flood prone areas in the reference scenario while this area only amounts to 34 km^2^ in the policy alternative. The small increase in flood risk in spite of the restrictions on building in flood risk areas under the policy alternative is a result of the increase of the flood prone area during the scenario period while the land use policies are based on the area currently under risk of flooding.

**Fig. 6:**
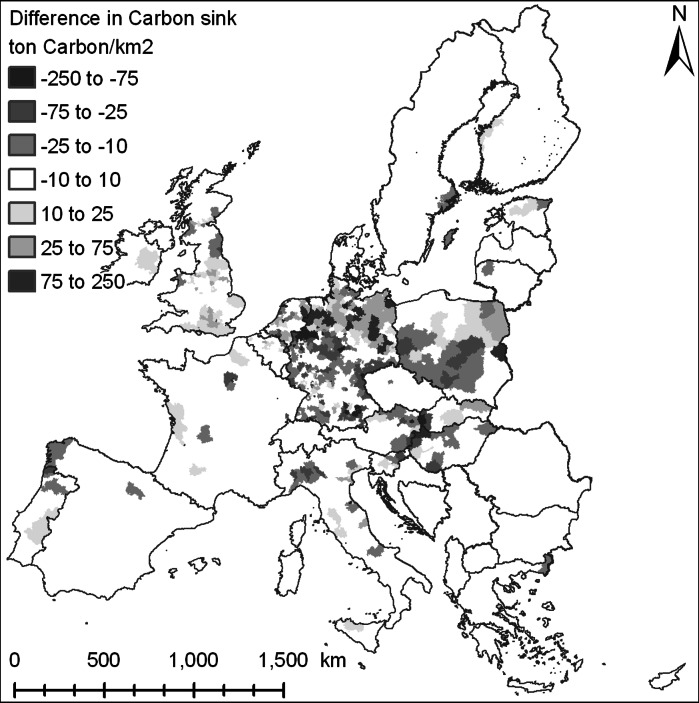
Comparison of difference in carbon sequestration (mean cumulative carbon sink per NUTS3-region in ton/km^2^ over the 2000–2030 period) between the reference scenario and the policy alternative. Positive values indicate higher values in the policy alternative

**Fig. 7:**
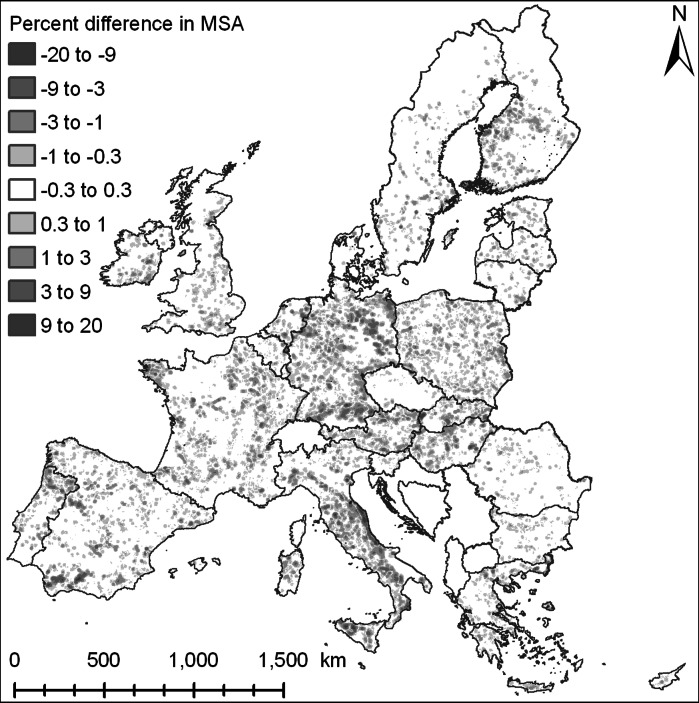
Local-level comparison of differences in mean species abundance between the reference scenario and policy alternative (weighted average within 10 km circular neighbourhood); positive values indicate higher MSA values in the policy alternative

The indicators of biodiversity and carbon sequestration are used to investigate if the adaptation measures lead to synergies with other ecosystem services. Figure 6 provides a map aggregated to administrative level of differences between the two scenarios in carbon sequestration at the level of the EU for 2030. Overall the differences resulting from the adaptation measures at the level of administrative regions are small. Many of the local impacts are compensated within the same administrative unit and therefore do not show in the map. At the same time the differences between the scenarios should not be ignored: depending on the region the impacts can be considerable. While in some regions synergies between adaptation measures and carbon sequestration are experienced other regions face negative trade-offs. Positive effects in Northern Germany are largely due to the policies that restrict the conversion of grasslands on peat soils. However, this leads to a lower rate of abandonment of arable land in Southern Germany, with corresponding lower carbon sequestration. Figure 7 shows that locally differences up to 20% in the mean species abundance values appear between the two scenarios. Where some regions show a strong synergy between the adaptation measures and biodiversity other regions show a negative tradeoff. In many cases the restrictions on intensive land uses in flood prone lands lead to positive effects on biodiversity. In a number of cases the incentives to convert arable land in upper catchments also has a positive effect on biodiversity while some of the unassigned areas face increasing land use pressures leading to biodiversity losses. It should be noted that the incentives for less intensive land use practices in upper catchments have a smaller effect on biodiversity than expected. Land abandonment is also taking place in these regions in the reference scenario which already fulfills some of the requirements in the policy alternative. Furthermore the success of the voluntary measures may be reduced as result to the increase in land use pressure due to land conversion restrictions in the lower parts of the catchments.

**Fig. 8:**
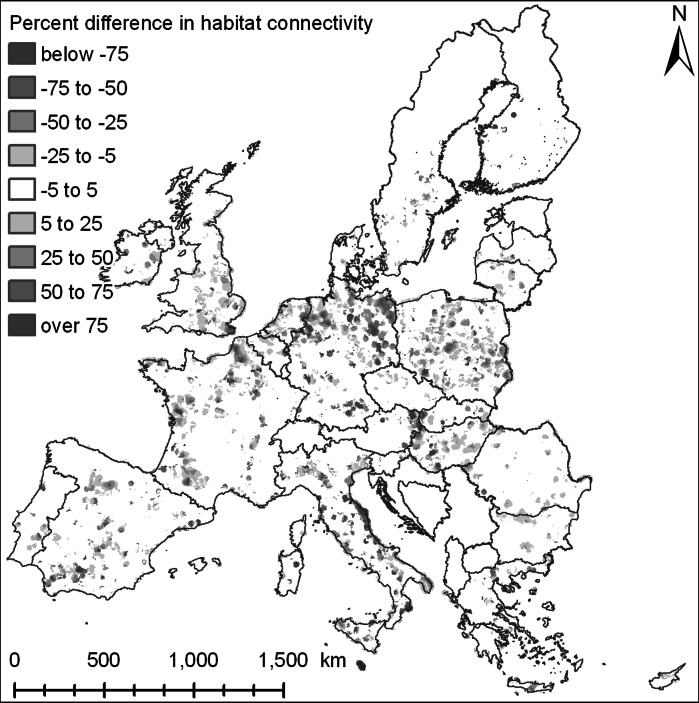
Differences in habitat connectivity between the reference scenario and policy alternative; negative values indicate a lower resistance to reach a habitat in the policy alternative

In general a decrease in the resistance to reach habitats (Fig. 8) is found at locations that also have an improvement in MSA value in Fig. 7. However, not all regions with an increase in MSA also have an improvement in habitat connectivity. The effects on habitat connectivity are strongest in regions that have very low habitat connectivity at present while at the same time adaptation measures lead to more extensive land uses that are providing opportunities to better connect existing habitats. The results also show that beneficial or negative impacts on habitat connectivity are only found at specific locations although adaptation measures are spread over large parts of Europe.

## Discussion

Adaptation to climate change consists of a wide range of measures related to different levels of governance. Measures range from local modifications of urban sewage systems to deal with higher peak flows to changes in national scale spatial planning policies and modifications in the common agricultural policy at EU level. In many of the measures land use plays a central role. Given that land use is central to the state of the environment and is linked to multiple economic sectors it is likely that policies in other fields will affect the effectiveness of adaptation measures while at the same time adaptation measures may provide synergies or trade-offs with other sectors. This paper presented a quantitative approach to analyze this mutual interaction between climate adaptation measures and other policy objectives in the context of a multi-scale analysis of land use dynamics. The results indicate that indeed the evaluated set of adaptation measures also impacts the other ecosystem services analyzed. In this paper only a small range of measures is analyzed and the impacts are assessed based on a limited set of indicators, only representing some of the ecosystem services provided in the study area. However, the approach allows for the evaluation of different scenarios and multiple impacts on ecosystem services (Kienast et al*.* 2009). The analysis is based on a straightforward top-down assessment of land use dynamics in which no feedbacks between the effects of the modified land allocation and the macro-economic conditions are assumed. This means that the implemented measures and their impacts do not influence the overall areas of the different land cover types. However, in reality such feedbacks are likely given that some of the incentives and regulations may affect land prices and, therefore, feedback on the trade and production conditions of the different countries (Verburg 2006). Currently the applied global scale models do not allow for incorporation of such feedbacks. The differences between the two scenarios simulated in this paper are, therefore, only resulting from differences in the spatial allocation of land use. Most of the impacts are found in the neighbourhood of the locations where the measures are implemented. However, the results indicate that restrictions to land use conversion at specific locations also lead to dynamics in land use elsewhere given the constant claims for land by the different sectors. Such spatial dynamics and teleconnections cause tradeoffs. While the reduction of intensity of land use in the flood plains leads to an improvement of the green infrastructure in these areas, more intensive land uses are now allocated outside these areas leading to negative impacts for biodiversity at those locations. Given that losses in biodiversity are not easily compensated by restoration elsewhere such off-site tradeoffs need to be accounted for when analyzing the synergies between climate change adaptation and biodiversity conservation. The results indicate that adaptation measures can, at least regionally, lead to a synergy with biodiversity conservation and climate regulation services through carbon sequestration. However, in regions where the land claims are high the measures frequently lead to strong tradeoffs in neighbouring regions. It would be interesting to test the sensitivity of a wider range of ecosystem services in terms of their synergies and tradeoffs. Raudsepp-Hearne et al. (2010) introduced the idea of identifying so-called ‘bundles’ of ecosystem services in which typical interactions between ecosystem services are embedded. While being appropriate for place-based research the concept could be extended by accounting for spatial interactions between ecosystem services using the approach used in this paper. Such research would also require a further analysis of sensitivity of the conclusions to the drivers of the model and the allocation procedure. Unfortunately global economic and integrated assessment models are only seldom analyzed in terms of sensitivity or uncertainty to major uncertainties in the driving factors and underlying model assumptions. The algorithms of the land use allocation models have been validated and tested in more detail (Messina et al*.* 2008; Pontius et al*.* 2008). For the European application of the Dyna-CLUE land allocation model the sensitivity to variation in macro-level drivers has been tested by Tabeau et al. (2010). Overall hot-spots of land change appeared to be relatively insensitive to variations in macro-scale drivers. Validation of the European allocation has not been made due to absence of consistent land cover data across multiple time periods to serve as a reference for such validation (Verburg et al*.* 2009a).

The effectiveness of the adaptation measures in reducing flood risk are not analyzed in this paper. The flood risk indicator solely indicates the exposed assets using a flood risk map that accounts for changes in climate conditions. The results make clear that in the absence of adaptation measures the urban area under flood prone conditions is likely to increase strongly. Changes in the hydrological circumstances as result of improved retention and reduced run-off in the upstream parts of the catchments are not accounted for and may reduce flood risk. Accounting for such changes would require a dynamic coupling of the land use simulations with a hydrological model. Such an approach was taken by Hurkmans et al. (2009) for analyzing the effects of changing climate and land use on extreme flow of the river Rhine. The authors indicated that the location of the land use changes within the catchment is very important for the effects on streamflow. In addition, they found that land use effects on streamflow are highly variable by sub-catchment. While extreme discharges in some sub-catchments were highly sensitive to changes in land use there were only modest effects in other sub-catchments. Therefore, an assessment of the effectiveness of measures to enhance the regulation of streamflow through land use requires a spatially explicit analysis.

The specification of the scenario options as an interactive process with the policy makers turned out to be a time-consuming process. However, during the specification an improved mutual understanding of the possible implications of the measures as well as an understanding of the capacities and limitations of assessment models to evaluate such measures was obtained. While initially defined in broad terms, the need for quantitative specification of the scenarios in the model provided a platform to discuss the more detailed implications of these policy themes for land use planning practices. In the end, the joint specification of the scenarios assisted the interpretation of the final modeling results because the policy makers had been involved in the process of specification which creates a feeling of ownership.

The analysis presented in this paper shows that integrative analysis of the tradeoffs and synergies of policy measures in a dynamic scenario context can benefit the targeting and selection of adequate policy measures. The analysis provides information to support discussion between different policy fields and allows to better explore the potential synergies and avoid unforeseen trade-offs. The process of scenario and model specification as a collaborative effort revealed the challenges of effective science-policy communication. While simple straightforward answers and assessments were preferred by the policy makers the discussion of the specification and implementation of scenario options in the model helped policy makers to understand the need for a clear specification of the broader policy objectives to be able to assess their impacts. The presentation of results in maps helped to understand the complexity of the outcomes. Trade-offs and synergies between adaptation measures and ecosystem service indicators are location and context dependent and land change assessments therefore do not always provide crisp and uniform answers to the questions of policy makers. As such, the science-policy interface emerged into a joint learning process in which the role of specific policies in complex human-environment interactions becomes clearer to both scientists and policy makers.

The approach presented in this paper is an example of operationalizing the ecosystem services approach to inform policy (Daily et al*.* 2009). The multi-sectoral and multi-scale characteristics of the results are an inherent characteristic of the ecosystem services approach and therefore require novel ways of science-policy interaction. The results indicate that although a generic adaptation strategy for Europe as a whole has a lot of benefit, it is at the local and regional level that the actual measures and implementations need to be designed in order to avoid unintended tradeoffs between services or conflicts with other policies. However, local measures should, at the same time, be analyzed in the context of regional and European impacts given the occurrence of spatial tradeoffs and the spatial distance between the locations where the measures are taken and the location of the beneficiaries of the adaptation measures and ecosystem services.

As land use is both a driver and result of human-environment interactions it provides a proper platform for discussing the way we can best adapt to changes in the earth system and secure the ecosystem services provided by the land.
